# External validation of two mpMRI-risk calculators predicting risk of prostate cancer before biopsy

**DOI:** 10.1007/s00345-022-04119-8

**Published:** 2022-08-08

**Authors:** Maximilian Pallauf, Fabian Steinkohl, Georg Zimmermann, Maximilian Horetzky, Pawel Rajwa, Benjamin Pradere, Andrea Katharina Lindner, Renate Pichler, Thomas Kunit, Shahrokh F. Shariat, Lukas Lusuardi, Martin Drerup

**Affiliations:** 1 Department of Urology, University Hospital Salzburg, Paracelsus Medical University, Muellner Hauptstraße 48, 5020 Salzburg, Austria; 2 Department of Urology, Comprehensive Cancer Center, Medical University of Vienna, Vienna, Austria; 3 Department of Radiology, Hospital St. Vinzenz Zams, Zams, Austria; 4 Team Biostatistics and Big Medical Data, IDA Lab Salzburg, Paracelsus Medical University, Salzburg, Austria; 5 Research and Innovation Management, Paracelsus Medical University, Salzburg, Austria; 6 Department of Urology, Medical University of Silesia, Zabrze, Poland; 7 Department of Urology, La Croix du Sud Hôpital, Quint Fonsegrives, France; 8 Department of Urology, Medical University of Innsbruck, Innsbruck, Austria; 9 Department of Urology, Weill Cornell Medical College, New York, NY USA; 10 Department of Urology, University of Texas Southwestern, Dallas, TX USA; 11 Institute for Urology and Reproductive Health I.M. Sechenov First Moscow State Medical University, Moscow, Russia; 12 Hourani Center for Applied Scientific Research, Al-Ahliyya Amman University, Amman, Jordan; 13 Department of Urology, Second Faculty of Medicine, Charles University, Prague, Czech Republic; 14 Karl Landsteiner Society, Vienna, Austria; 15 Department of Urology, Hospital Barmherzige Brüder Salzburg, Salzburg, Austria

**Keywords:** Prostate cancer, Risk calculators, Nomogram, Prostate biopsy

## Abstract

**Purpose:**

Risk calculators (RC) aim to improve prebiopsy risk stratification. Their latest versions now include multiparametric magnetic resonance imaging (mpMRI) findings. For their implementation into clinical practice, critical external validations are needed.

**Methods:**

We retrospectively analyzed the patient data of 554 men who underwent ultrasound-guided targeted and systematic prostate biopsies at 2 centers. We validated the mpMRI-RCs of Radtke et al. (RC-R) and Alberts et al. (RC-A), previously shown to predict prostate cancer (PCa) and clinically significant PCa (csPCa). We assessed these RCs’ prediction accuracy by analyzing the receiver-operating characteristics (ROC) curve and evaluated their clinical utility using Decision Curve Analysis (DCA), including Net-Benefit and Net-Reduction curves.

**Results:**

We found that the Area Under the ROC Curve (AUC) for predicting PCa was 0.681 [confidence interval (CI) 95% 0.635–0.727] for RC-A. The AUCs for predicting csPCa were 0.635 (CI 95% 0.583–0.686) for RC-A and 0.676 (CI 95% 0.627–0.725) for RC-R. For example, at a risk threshold of 12%, RC-A needs to assess 334 and RC-R 500 patients to detect one additional true positive PCa or csPCa patient, respectively. At the same risk threshold of 12%, RC-A only needs to assess 6 and RC-R 16 patients to detect one additional true negative PCa or csPCa patient.

**Conclusion:**

The mpMRI-RCs, RC-R and RC-A, are robust and valuable tools for patient counseling. Although they do not improve PCa and csPCa detection rates by a clinically meaningful margin, they aid in avoiding unnecessary prostate biopsies. Their implementation could reduce overdiagnosis and reduce PCa screening morbidity.

**Supplementary Information:**

The online version contains supplementary material available at 10.1007/s00345-022-04119-8.

## Introduction

Implementing multiparametric Magnetic Resonance Imaging (mpMRI) into the PCa diagnosis pathway has been essential in refining prostate cancer (PCa) detection. Visualization of prostate areas suspicious for cancer combined with targeted biopsies improved the detection of clinically significant cancer while simultaneously reducing the number of biopsies [1].

Nevertheless, we are far from having the optimal triage test. Too many clinically significant prostate cancers (csPCa) are overlooked, too many unnecessary biopsies are performed, and too many insignificant PCas are detected [1]. This is because prostate mpMRI is generally working as a rule-out test. Its negative predictive value (NPV) is 80–85%, whereas its positive predictive value (PPV) is 17–75% and is highly dependent on the mpMRI quality and the radiologist’s experience [2].

Risk calculators (RC) are an alternative method to improve PCa diagnostics. Promoted in the pre-MRI era, they calculate the risk for PCa and csPCa through the combination of various clinical variables. The European Randomized Study of Screening for Prostate Cancer (ERSPC) [3] and the Prostate Cancer Prevention Trial (PCPT) [4] have presented the most well-known RCs. However, while showing good discrimination accuracy on internal validation, external validation cohorts revealed a lack of reproducibility [5–8].

Most recently, various research groups implemented mpMRI findings into the ERSPC-RC. Among these, the RCs of Radtke et al. [9] and Alberts et al. [10] showed the most promising results in internal and external validation studies [11–18]. As mpMRI quality is highly dependent on the radiologist’s experience, among other factors, it is unclear whether these RCs accurately predict the risk of PCa and csPCa when including mpMRI reports not performed by dedicated uro-radiologists.

Therefore we externally validated the RCs by Radtke et al. [9] (RC-R) and Alberts et al. [10] (RC-A) on a “real-life” scenario multicenter patient cohort, including mpMRI reports of radiologists with unknown experience with prostate cancer. This study aims to assess the RCs’ robustness in predicting PCa and csPCa and evaluate their value for general clinical practice.

## Materials and methods

### Patient data

We retrospectively reviewed all patients who underwent ultrasound-guided fusion biopsy of the prostate at two university hospitals between 01/2015 and 01/2017. All patients received targeted and systematic prostate biopsies, either by a transrectal or transperineal approach. According to the treating physician’s preference, the targeted biopsy was performed either by cognitive or software-assisted fusion.

Internal specially trained uro-radiologists and external practicing radiologists rated the mpMRI findings. No information on the external practicing radiologists’ skills and experience can be given. No internal validations of the external radiologists’ mpMRI reports were performed.

We included patients with previous negative biopsy findings only and if all of the following information was present: patient age (years); prostate-specific antigen (PSA) level (ng/dl); prostate volume (milliliter); digital rectal examination report; Prostate Imaging—Reporting and Data System (PI-RADS) score; prostate biopsy pathology report; radical prostatectomy pathology report (only if performed in one of the participating centers); surgical report.

### Definition of clinically significant prostate cancer

In concordance with the RCs under validation, we defined csPCa as ISUP grade group two and above. This consideration is supported by the definitions of the PRECISION study group [19]. If the radical prostatectomy pathology report revealed contrary findings to the biopsy pathology report, ISUP grade group two instead of one, the PCa was defined as clinically significant for validation purposes.

### Risk calculations

The individual PCa and csPCa risks were calculated for every patient using both RCs. For this purpose, Radtke et al. [9] provided the formula of RC-R. The input variables were adapted to the RC’s specifications. Alberts et al. [10] provided RC-A’s patient results blinded to the pathology reports.

### Statistics

The RCs’ diagnostic accuracy was evaluated by estimating Receiver Operating Characteristic (ROC) curves and calculating corresponding 95% confidence intervals (CI) for the whole dataset. We also performed a subgroup analysis and created ROCs based on the PI-RADS classification (III, IV, V). Further, we performed a decision-curve analysis (DCA) and calculated Net-Benefit and Net-Reduction curves. In addition, we calculated the true-positive and true-negative net benefit (TP-NB; TN-NB) at specific risk thresholds and calculated the number needed to treat to detect one additional true-positive or true-negative patient. DCAs were adjusted to the prevalence of PCa and csPCa in our patient cohort. The two-sided significance level was set to 5% (accordingly, two-sided 95% confidence levels were calculated). All calculations were performed using the statistical software R version 3.5.1, using pROC and rmda packages.

### Ethics

  The Institutional Review Boards of both centers approved this study.

## Results

### Patient characteristics

We included 554 patients, 101 from Center A and 453 from Center B. The median age was 64.5 years [interquartile range (IQR) 58.6–70.7], the median prostate volume 46 ml (IQR 35–65), and the median PSA level 7.0 ng/ml (IQR 4.8–10.2). Digital rectal examination was suspicious in 8.3% of the patients, and most patients had been diagnosed with a PI-RADS IV lesion (71.7%). We took a median of 15 biopsies (IQR 15–15). The prevalence for PCa and csPCa were 32% and 23.8%, respectively.

Table 1 provides a detailed overview of our patient cohort and RC-R and RC-A development cohorts published in the original manuscripts [9, 10].

**Table 1:** This table gives an overview of the patient characteristics of the RCs’ development cohorts and the external validation cohort

	Study cohort	RC-R	RC-A
Number of patients all	554	1159	1353
Number of patients repeat-biopsy	554	489	802
Age—years	64.5 (58.6–70.7)	65 (60–71)	66.0 (60.0–71.0)
Digital rectal examination positive	46 (8.3%)	23.0%	22.5%
PSA (ng/dl)	7.0 (4.8–10.2)	N/A	8.7 (6.1–12.9)
Prostate volume (ml)	46 (35–65)	45 (33–64)	50 (36–70)
PI-RADS II	12 (2.2%)	15%	17.7%^1^
PI-RADS III	49 (8.8%)	33%	18.5%
PI-RASD IV	397 (71.7%)	32%	39.7%
PI-RADS V	96 (17.3%)	20%	24.2%
Cognitive fusion biopsy	77 (13.9%)	0.0%	3.2%
Software assisted fusion biopsy	477 (86.1%)	100.0%	96.8%
Number of biopsies	15 (15–15)	27 (24–29)	N/A
Number of biopsies positive for PCA	2 (0–5)	N/A	N/A
PCa–all patients	177 (32.0%)	63%	51.2%
PCa–repeat-biopsy	177 (32.0%)	64%	N/A
csPCa–all patients	132 (23.8%)	42%	35.7%
csPCa–repeat-biopsy	132 (23.8%)	N/A	N/A
AUC PCa			0.79
AUC csPCa		0.81	0.85

*n* (%), Median (IQR)

^1^PI-RADS I + II

### External validation of risk calculators

#### ROC-curve analysis

**Fig. 1:**
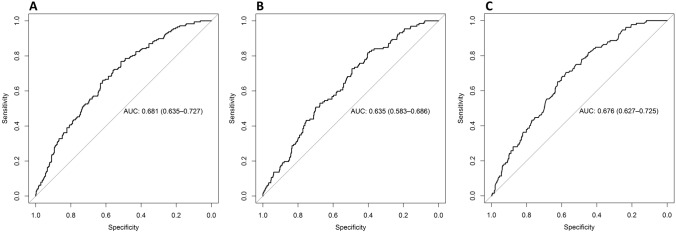
This figure gives the ROC analyses for RC-A predicting the risk for PCa (**A**) and csPCa (**B**), and for RC-R predicting the risk for csPCa (**C**). The estimated AUCs, including 95% CIs, are given

For predicting PCa, the area under the ROC curve (AUC) for RC-A was 0.681 [confidence interval (CI) 95% 0.635–0.727], (Fig. 1A). For predicting csPCa, the AUCs were 0.635 (CI 95% 0.583–0.686) for RC-A and 0.676 (CI 95% 0.627–0.725) for RC-R, (Fig. 1B, C). The comparison of the csPCa ROC curves showed no significant difference.

Subgroup analyses based on PI-RADS classification showed a trend towards a better diagnostic accuracy in PI-RADS III lesions for both RCs. However, the low number of patients in the subgroup analyses and the wide CIs prevent further interpretation of the data.

ROC curves grouped by PI-RADS classification are shown in Supplementary Figs. 1, 2.

#### Decision curve analysis

For assessing the RCs’ clinical benefit, DCA, including calculations of Net-Benefit and Net-Reduction curves, were performed. We decided on the best RC in terms of the AUC point estimator for PCa (RC-A) and csPCa (RC-R).

##### RC-A – PCa

The TP-NB of RC-A for detecting PCa was comparable to the “treat all” strategy at a risk threshold of 4% [TP-NB 0.291 (CI 95% 0.291–0.291)], but it was superior at risk thresholds of 12% and beyond. For example, at the risk thresholds of 12% and 20%, the TP-NBs were 0.230 (CI 95% 0.228–0.232) and 0.161 (CI 95% 0.153–0.169). Setting the risk thresholds for RC-A to 12% and 20%, 334 and 86 patients, respectively, have to be assessed to detect one additional true positive PCa patient.

The TN-NB of RC-A for detecting PCa showed no benefit at a risk threshold of 4% [TN-NB 0.000 (– 0.000–0.000)], but it was superior to the "treat none" strategy at risk thresholds of 4% and beyond. For example, at the risk thresholds of 12% and 20%, the TN-NBs were 0.023 (CI 95% 0.011–0.036) and 0.047 (CI 95% 0.009–0.078). Setting the risk thresholds for RC-A to 12% or 20%, six patients have to be assessed to detect one additional true-negative PCa patient.

**Fig. 2:**
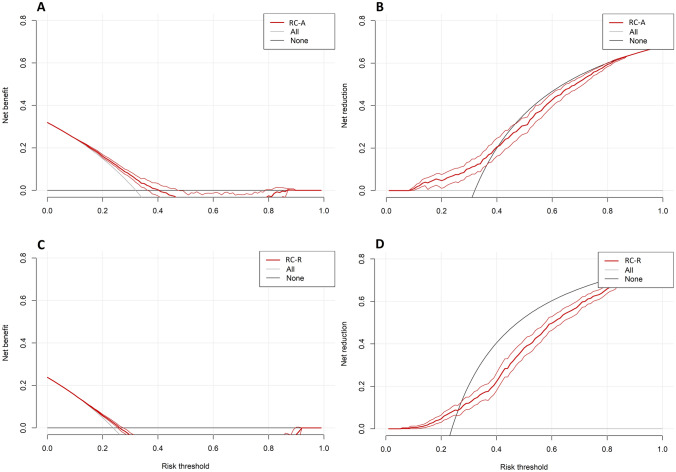
This figure gives the Net-Benefit and Net-Reduction curves for RC-A predicting PCa (**A**, **B**) and for RC-R predicting csPCa (**C**, **D**). For the Net-Benefit curves (**A**, **C**), the *x*-axes give the NB, and the *y*-axes give the specific risk threshold probability. Further, the *y*-axes equal the treatment strategy “treat-none”, and the grey lines indicate “treat all”. The Net-Benefit curves are given, including 95% CIs. For the Net-Reduction curves (**B**, **D**), the *x*-axes give the Net-Reduction in interventions per 100 patients, and the *y*-axes give the specific risk threshold probability. Further, the *y*-axes equal the treatment strategy “treat-all”, and the black lines indicate “treat none”. The Net-Reduction curves are given, including 95% CIs

Figure 2A, B shows RC-A’s Net-Benefit and Net-Reduction Curve.

##### RC-R – csPCa

The TP-NB of RC-R for detecting csPCa was not different from the “treat all” strategy at a risk threshold of 4% [TP-NB 0.206 (CI 95%0.206–0.206)], but it was superior at risk thresholds of 12% and beyond. For example, at the risk thresholds of 12% and 20%, the TP-NBs were 0.136 (CI 95% 0.135–0.137) and 0.059 (CI 95% 0.055–0.065). Setting the risk thresholds for RC-R to 12% and 20%, 500 and 91 patients, respectively, have to be assessed to detect one additional true-positive csPCa patient.

The TN-NB of RC-R for detecting csPCa showed no benefit at a risk threshold of 4% [TN-NB 0.000 (CI 95% 0.000–0.000)], but it was superior to the "treat none" strategy at risk thresholds of 4% and beyond. For example, at the risk thresholds of 12% and 20%, the TN-NBs were 0.009 (CI 95% 0.002–0.018) and 0.047 (CI 95% 0.031–0.067). Setting the risk thresholds for RC-R to 12% and 20%, 16 and six patients, respectively, have to be assessed to detect one additional true-negative csPCa patient.

Figure 2C, D shows RC-R’s Net-Benefit and Net-Reduction Curve.

## Discussion

This manuscript is the first external validation study testing RC-R and RC-A in two centers within a “real-life” scenario. Contrary to previously published validation studies [11–18], our validation cohort had significant heterogeneity. We included patients with mpMRIs not performed by dedicated uro-radiologists but multiple radiologists with differing degrees of expertise. Furthermore, prostate biopsies were performed either by a transrectal or transperineal approach under local or general anesthesia, and different software-assisted fusion biopsy systems were used. The lack of standardization between multiple centers, which is common outside of prospective trials, might have reduced the mpMRI-RCs’ discrimination accuracy. However, testing the mpMRI-RCs in a “real-life” setting is crucial for determining the models’ robustness, generalizability, and clinical benefit [20].

Our cohort showed significant heterogeneity, but patient characteristics also differed substantially from the cohorts used in the original manuscripts for RC development [9, 10]. For example, suspicious findings on digital rectal examination were less common in our cohort (8.3% vs. 23% RC-R and 22% RC-A), and the distribution of PI-RADS varied considerably. PI-RADS lesions IV and V were more commonly detected in our cohort (89%) than in the cohorts of RC-R (52%) and RC-A (63.9%). Furthermore, fewer prostate biopsy cores were taken in our cohort, with a median of 15 (– 15–15) compared to 27 (IQR 24–29) for the cohort of RC-R. For the cohort of RC-A, this data was not given in the original manuscript. Most importantly, our cohort included patients with a previous negative systematic biopsy (100%), whereas this was only partly the case for the cohorts of RC-R (42%) and RC-A (59%). These differences in relevant patient characteristics might explain our cohort’s low PCa and csPCa prevalence and the reduced diagnostic accuracy found in our validation study. However, the validation of RC-R and RC-A using a cohort with substantially different patient characteristics strengthens their robustness and general validity.

We found that DCA adjusted for differences in patient PCa and csPCa prevalence revealed important findings. First, the RCs’ TP-NB was marginally superior to the “treat-all” strategy at various risk thresholds. At a risk threshold of 12%, which means that the benefit of detecting one additional PCa or csPCa patient is valued approximately eight times more than the negative consequences of performing an unnecessary biopsy, 334 to 500 patients have to be tested for the respective RC to be of value. The high number needed to treat limit the RCs’ utility in clinical practice to improve PCa and csPCa detection. Second, the RCs’ TN-NB was superior to the "treat-none" strategy at risk thresholds of 4%, 12%, and 20%. At a risk threshold of 12%, which means the negative consequences of missing one PCa or csPCa are valued approximately 8 times more than the negative consequences of performing an unnecessary biopsy, 6 to 16 patients only have to be tested for the respective RC to be of value. The low number needed to treat highlight the RCs’ benefit for reducing unnecessary prostate biopsies.

Our external validation study showed that RC-R and RC-A strengthen the value of mpMRI as a rule-out test [2]. Further, they highlight that combining mpMRI-findings with clinical parameters can aid in balancing out differences in mpMRI quality and the reader’s experience. Interestingly our subgroup analysis, which stratified patients based on PI-RADS classification, revealed the best discrimination accuracy for PI-RADS III lesions. Although this result must be interpreted cautiously due to the small sample size, it is of importance as PI-RADS III is a heterogeneous category with variable risk of PCa and csPCa presence. Hence PCa rates in this population are approximately 17% only, the number of unnecessary biopsies is high [2]. The RCs may give treating physicians more confidence in avoiding biopsies in this group.

Our study has some limitations. First, data were collected retrospectively, leading to a risk of selection bias. Second, the centers did not participate equally in patient recruitment, and the mpMRI pathway and biopsy strategy were not standardized. Nevertheless, the heterogeneity of our patient cohort strengthens the study findings as we tested RC-R and RC-A in a “real-life” scenario. Third, patients with negative prostate biopsy findings were followed up with external urologists. Therefore, follow-up data is missing, and no information on undetected PCa and csPCa can be given.

## Conclusions

The mpMRI-RCs, RC-R and RC-A, are robust and valuable tools for patient counseling. Although they do not improve PCa and csPCa detection rates y a clinically meaningful margin, they aid in avoiding unnecessary prostate biopsies. Their implementation could reduce overdiagnosis and further reduce the morbidity of PSA screening.

## Supplementary Information

Below is the link to the electronic supplementary material.

Supplementary file1 **Supplementary Fig. 1** This figure gives the ROC analyses for RC-A predicting the risk for PCa within PI-RADS III (**A**), IV (**B**), and V (**C**) lesions only. The calculated AUCs, including 95% CIs, are given (TIF 870 KB)

Supplementary file2 **Supplementary Fig. 2** This figure gives the ROC analyses for RC-R predicting the risk for csPCa within PI-RADS III (**A**), IV (**B**), and V (**C**) lesions only. The calculated AUCs, including 95% CIs, are given (TIF 885 KB)

## Abbreviations

AUC: Area under the curve
CI: Confidence intervals
csPCa: Clinically significant prostate cancer
DCA: Decision Curve Analysis
ERSPC: European Randomized Study of Screening for Prostate Cancer
IQR: Interquartile range
mpMRI: Multiparametric Magnetic Resoncance Imaging
NPV: Negative predictive value
PCa: Prostate cancer any grade
PCPT: Prostate Cancer Prevention Trial
PI-RADS: Prostate Imaging—Reporting and Data System
PPV: Positive predictive value
PSA: Prostate-specific antigen
RC: Risk calculator
RC-A: Risk calculator by Alberts et al.
RC-R: Risk calculator by Radtke et al.
TN-NB: True negative net benefit
TP-NB: True positive net benefit
