# Supplementary figures and images for: External validation of two mpMRI-risk calculators predicting risk of prostate cancer before biopsy
Source: World J Urol. 2022 Aug 8;40(10):2451–7. doi: 10.1007/s00345-022-04119-8 (PMC9512729; doi:10.1007/s00345-022-04119-8)

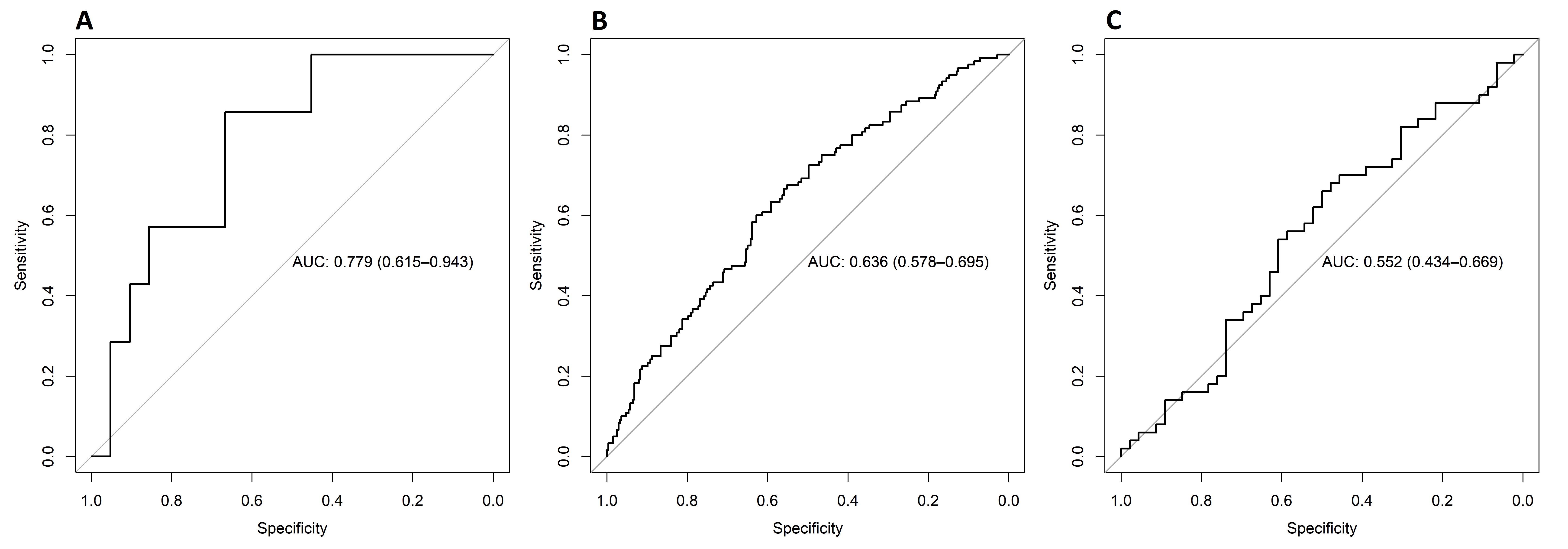

Supplement: Supplementary file 1 — Supplementary file1 Supplementary Fig. 1 This figure gives the ROC analyses for RC-A predicting the risk for PCa within PI-RADS III (A), IV (B), and V (C) lesions only. The calculated AUCs, including 95% CIs, are given (TIF 870 KB) [file 345_2022_4119_MOESM1_ESM.tif]

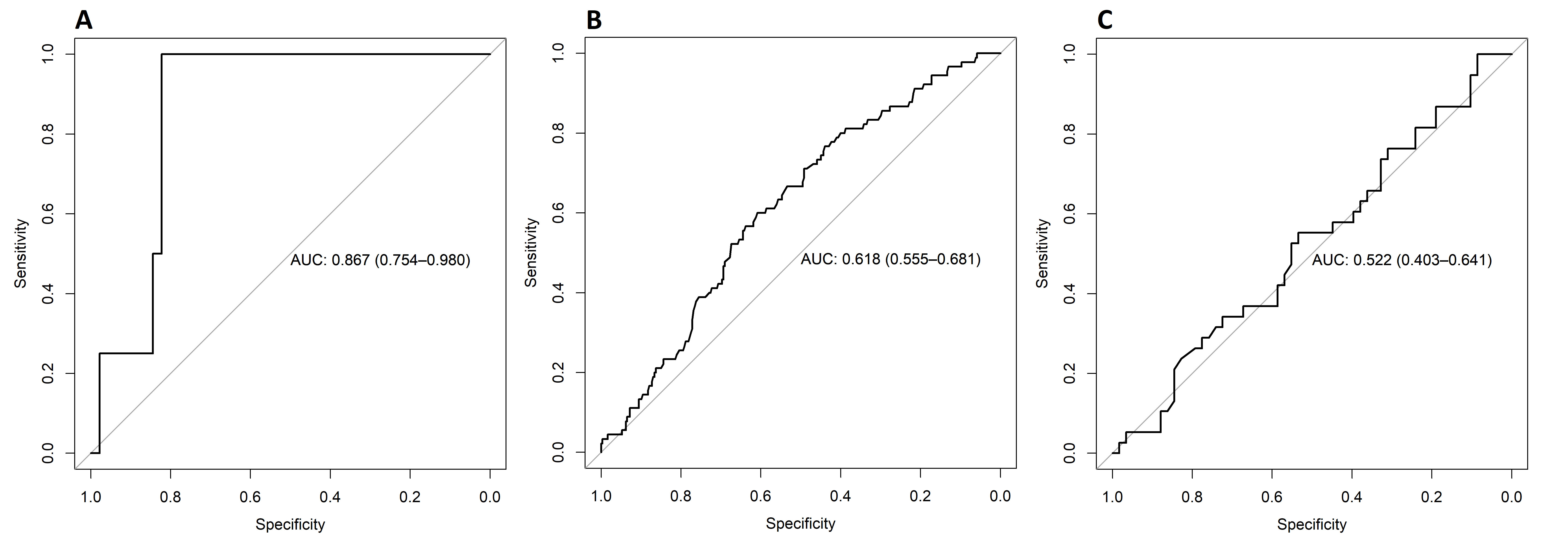

Supplement: Supplementary file 2 — Supplementary file2 Supplementary Fig. 2 This figure gives the ROC analyses for RC-R predicting the risk for csPCa within PI-RADS III (A), IV (B), and V (C) lesions only. The calculated AUCs, including 95% CIs, are given (TIF 885 KB) [file 345_2022_4119_MOESM2_ESM.tif]
